# The Acetyl Bromide Method Is Faster, Simpler and Presents Best Recovery of Lignin in Different Herbaceous Tissues than Klason and Thioglycolic Acid Methods

**DOI:** 10.1371/journal.pone.0110000

**Published:** 2014-10-16

**Authors:** Flavia Carolina Moreira-Vilar, Rita de Cássia Siqueira-Soares, Aline Finger-Teixeira, Dyoni Matias de Oliveira, Ana Paula Ferro, George Jackson da Rocha, Maria de Lourdes L. Ferrarese, Wanderley Dantas dos Santos, Osvaldo Ferrarese-Filho

**Affiliations:** 1 Department of Agronomy, State University of Maringá, Maringá, Paraná, Brazil; 2 Department of Biochemistry, State University of Maringá, Maringá, Paraná, Brazil; 3 Brazilian Bioethanol Science and Technology Laboratory, Campinas, São Paulo, Brazil; University of Vigo, Spain

## Abstract

We compared the amount of lignin as determined by the three most traditional methods for lignin measurement in three tissues (sugarcane bagasse, soybean roots and soybean seed coat) contrasting for lignin amount and composition. Although all methods presented high reproducibility, major inconsistencies among them were found. The amount of lignin determined by thioglycolic acid method was severely lower than that provided by the other methods (up to 95%) in all tissues analyzed. Klason method was quite similar to acetyl bromide in tissues containing higher amounts of lignin, but presented lower recovery of lignin in the less lignified tissue. To investigate the causes of the inconsistencies observed, we determined the monomer composition of all plant materials, but found no correlation. We found that the low recovery of lignin presented by the thioglycolic acid method were due losses of lignin in the residues disposed throughout the procedures. The production of furfurals by acetyl bromide method does not explain the differences observed. The acetyl bromide method is the simplest and fastest among the methods evaluated presenting similar or best recovery of lignin in all the tissues assessed.

## Introduction

Lignin is a complex heteropolymer that primarily consists of *p*-hydroxyphenyl (H), guaiacyl (G), and syringyl (S) units formed by the oxidative coupling of *p*-coumaryl, coniferyl, and sinapyl alcohols, respectively, which are products of the phenylpropanoid pathway [1]. After complete deposition in the secondary cell wall, lignin provides a coating for cellulose-hemicellulose microfibrils, thus ensuring stiffness, strength, and impermeability for the lignified tissues. These properties protect the energetic polysaccharides against attack from pathogens and herbivores [2].

Due to the importance of lignin for water transport in plants, mechanical resistance of grains, the textile and paper industries, and as a major hindrance in the generation of bioethanol [2], [3], there is a growing interest in improving the assays used for its quantification. Several methods have been described to measure lignin levels in biomass samples [4]–[8]. However, there is no universal method to determine lignin. This is a grave drawback because each technique can give different results for a same sample.

**Figure 1:**
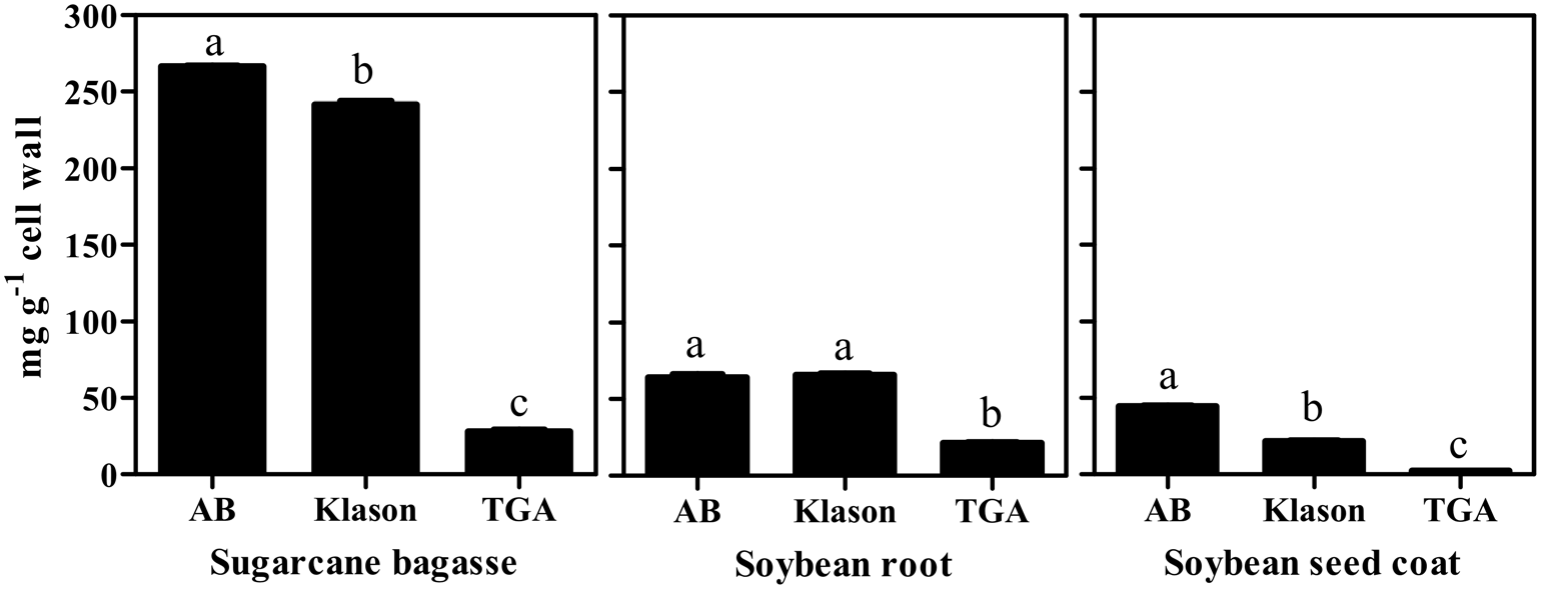
Lignin content of sugarcane bagasse, soybean root and soybean seed coat determined by the acetyl bromide, Klason lignin and thioglycolic acid methods. AB: acetyl bromide method; TGA: thioglycolic acid method. Mean values ± SE (N = 4) marked with different letters are significantly different (P≤0.05, Scott-Knott test).

The acetyl bromide, thioglycolic acid, and Klason are the more common methods used to quantify lignin [9]. The two first methods are based on solubilization of lignin and determination of absorbance values at 280 nm. Initially developed to provide a sensitive method for small samples of forage plants, the acetyl bromide protocol is based on the formation of acetyl derivatives in non substituted OH groups and bromide replacement of the α-carbon OH groups to produce a complete solubilization of lignin under acidic conditions. Because it is not a direct method, an overestimation of the lignin content can occur due to the oxidative degradation of structural polysaccharides (*e.g.* xylans) during the incubation of the cell wall with the acid solution [10]. By its time, thioglycolic acid method is based in the formation of thioethers of benzyl alcohol groups found into the lignin, resulting in solubilization of this polymer under alkaline conditions. However, due to the specificity of the reaction with ether bond types of lignin, the thioglycolic acid method can underestimate the lignin content [11]. In addition, during the precipitation steps, a fraction of soluble lignin can remain in solution, thereby resulting in an underestimation of the total lignin content [9]. The Klason method is a gravimetric assay used for the direct quantification of lignin. In this method, an insoluble lignin fraction is extracted from the plant tissues after digestion with 72% sulfuric acid followed by partial solubilization of cell wall polysaccharides. However, compounds such as proteins and non-extracted polysaccharides can be measured together with lignin, resulting in an overestimation of the lignin content^9^. On the other hand, a considerable fraction of soluble lignin cannot be measured resulting in underestimation of lignin [12]. Despite of the inconsistencies reported [9]–[11] these methodologies are quite consolidated for use in wood biomasses. However, when coping with herbaceous plants in which the amount of lignin might be quite lower and sugar content quite higher than in wood, these inconsistencies among the methods might be amplified. There are few reports comparing the spectrophotometric and gravimetric protocols for lignin quantification, and they are not conclusive [11], [13], [14]. This fact raises reasonable doubts about the reliability of current methods, and it has gained the interest of many researchers. Furthermore, choosing the appropriate method to determine lignin in a specific test is still an open question. Therefore, we compared the total lignin content in sugarcane bagasse, soybean root, and soybean seed coat using the acetyl bromide, thioglycolic acid and Klason methods (Fig. 1). We also evaluated the monomeric composition of lignin in the contrasting tissues, as well as the production of furfurals in order to investigate the causes of the inconsistencies observed among the methods.

The plant materials were selected based on their contrasting lignin content and monolignol composition, as well as their economic and scientific relevance. Sugarcane presents a type II cell wall while soybean have type I cell wall. Both are major global crops [15], [16]. The amount of lignin is conversely related with cellulose digestibility hindering the nutritional power and cellulosic ethanol yield [17]. In soybean, lignin provides protection against attack from pathogens and increases the mechanical resistance of the grains during transport and processing [18].

## Materials and Methods

### General procedures

To determine the amount of lignin in the sugarcane bagasse, dried samples were obtained from the National Laboratory of Science and Technology of Bioethanol (CTBE, Brazil). For root lignin content determination, soybean (*Glycine max* L. Merrill) seeds were surface-sterilized with 2% sodium hypochlorite for 5 min, rinsed extensively with deionized water, and then germinated in the dark at 25°C on two sheets of filter paper moistened with water. After three days, the roots were excised and dried in an oven at 60°C until a constant weight was achieved. For seed coat lignin content determination, soybean seeds were initially immersed in water for 12 h to separate the seed coats from the cotyledons. Then, the seed coats were dried in an oven at 60°C until a constant weight was achieved.

### Protein-free cell wall preparation

All protocols used to quantify lignin were carried out after exhaustive preparation of the tissues to exclude protein and other UV-absorbing materials. This removal was essential to avoid the measurement of these constituents together with lignin at 280 nm. Dry samples (0.3 g) were homogenized in 50 mM potassium phosphate buffer (7 ml, pH 7.0) using a mortar and pestle and then transferred into a centrifuge tube [19]. The pellet was centrifuged (1,400×*g*, 5 min) and washed by successive stirring and centrifugation as follows: two times with phosphate buffer (pH 7.0; 7 ml), three times with 1% (v/v) Triton X-100 in pH 7.0 buffer (7 ml), two times with 1 M NaCl in pH 7.0 buffer (7 ml), two times with distilled water (7 ml), and two times with acetone (5 ml). The pellet was dried in an oven (60°C, 24 h) and cooled in a vacuum desiccator. The dry matter obtained was defined as the protein-free cell wall fraction.

### Quantification of lignin by the thioglycolic acid method

Protein-free cell wall samples (0.1 g) were placed into a screw-cap centrifuge tube containing the reaction mixture (1.2 ml of thioglycolic acid and 6 ml of 2 M HCl) and heated (95°C, 4 h). After cooling at room temperature, the sample was centrifuged (1,400×*g*, 5 min), and the supernatant was discarded. The pellet contained the LTGA. The pellet was washed three times with distilled water (7 ml), and the LTGA was extracted by shaking (30°C, 18 h, 115 oscillation min^−1^) in 0.5 M NaOH (6 ml). After centrifugation (1,400×*g*, 5 min), the supernatant was reserved and the pellet was washed again with 0.5 M NaOH (3 ml). The supernatant were mixed and acidified with concentrated HCl (1.8 ml). After precipitation (4°C, overnight), the LTGA was recovered by centrifugation (1,400×*g*, 5 min) and washed twice with distilled water (7 ml). The pellet was dried at 60°C, then dissolved in 0.5 M NaOH, and diluted to yield an appropriate absorbance for spectrophotometric determination at 280 nm. The lignin content was expressed as mg LTGA g^−1^ dry weight [20]. A standard curve for lignin (alkali, 2-hydroxy-proyl ether, Aldrich 37,096-7) was generated, and the absorptivity (ε) value obtained was 18.31 g^−1 ^L cm^−1^. The results were expressed as mg LTGA g^−1^ cell wall ± Standard Error (SE).

### Quantification of lignin by the acetyl bromide method

Protein-free cell wall sample (20 mg) was placed into a screw-cap centrifuge tube containing 0.5 ml of 25% acetyl bromide (v/v in glacial acetic acid) and incubated at 70°C for 30 min. After complete digestion, the sample was quickly cooled in an ice bath, and then mixed with 0.9 ml of 2 M NaOH, 0.1 ml of 5 M hydroxylamine-HCl, and a volume of glacial acetic acid sufficient for complete solubilization of the lignin extract (4 ml for soybean tissues or 6 ml for sugarcane bagasse). After centrifugation (1,400×*g*, 5 min), the absorbance of the supernatant was measured at 280 nm [21]. A standard curve was generated with alkali lignin (Aldrich 37, 096-7) and the absorptivity (ε) value obtained was 22.9 g^−1 ^L cm^−1^. The results were expressed as mg lignin g^−1^ cell wall.

### Quantification of lignin by the Klason method

Protein-free cell wall sample (1.0 g) was digested (72% H_2_SO_4_, 47°C, 7 min) under vigorous stirring. After complete digestion, the sample was autoclaved (121°C, 1 atm, 30 min), followed by filtration through filter paper to separate the soluble and insoluble fractions [22]. The soluble fraction was measured at 280 nm and 215 nm, and the lignin content was calculated by the following formula: S = (4.53 *A*_215_–*A*_280_)/300. This formula is the simultaneous resolution of two equations: A_280_ = 0.68 F+18 S and A_215_ = 0.15 F+70 S, where A_280_ is the absorbance value at 280 nm, A_215_ is the absorbance value at 215 nm, F is the furfural concentration (g L^−1^), and S is the soluble lignin concentration (g L^−1^). The values 0.68 and 0.15 are the molar absorptivities of furfurals at 280 and 215 nm, respectively. The values 18 and 70 are the molar absorptivities of soluble lignin at 280 and 215 nm, respectively [23]. Ash content of all samples was determined by combustion of the insoluble fraction at 550°C for 4 hours. Lignin from the insoluble fraction was calculated by the difference in the weight of the dry mass and the total ash for each sample [22]. The lignin content was determined by the sum of the insoluble and soluble lignin and was expressed as mg g^−1^ cell wall.

### Lignin monomer composition

The oxidation of alkaline nitrobenzene was used to determine the lignin monomer composition [24]. This technique causes the degradation of lignin, resulting in the formation of *p*-hydroxybenzaldehyde from the H unit, vanillin from the G unit, and syringaldehyde from the S unit. The protein-free cell wall sample (50 mg) previously obtained was sealed in a Pyrex ampoule containing 1 ml of 2 M NaOH and 1 ml of nitrobenzene prior to heating at 170°C for 90 min with occasional shaking during the reaction. The sample was then cooled at room temperature, washed twice with chloroform, acidified to pH 2 with 2 M HCl, and then extracted twice with chloroform. The organic extracts were combined, dried, and suspended in 1 ml of a mixture of methanol and 4% acetic acid in water (20∶80, v/v). The sample was filtered through a 0.45-µm disposable syringe filter (Hamilton Co., Nevada, USA), and a 20 µl aliquot was analyzed in a high performance liquid chromatography (HPLC) system (Shimadzu 10AVP, Tokyo, Japan) equipped with an LC-10AD pump, a Rheodyne injector, an SPD-10A UV detector, a CBM-101 Communications Bus Module, and a Class-CR10 workstation system. A reversed-phase Shimpack CLC-ODS column (150 mm×4.6 mm, 5 µm), protected with an equivalent pre-column, was used at 30°C. The mobile phase consisted of a mixture of methanol and 4% acetic acid in water (20∶80, v/v) and had a flow rate of 1.2 ml min^−1^ for an isocratic run of 20 min. Quantification of the monomer aldehyde products (*p*-hydroxybenzaldehyde, vanillin, and syringaldehyde) released by nitrobenzene oxidation was performed at 290 nm using the corresponding standards. The results were expressed as mg monomer g^−1^ cell wall.

### Quantification of furfurals

After the reaction with acetyl bromide, the samples were filtered through a 0.45-µm disposable syringe filter (Hamilton Co., Nevada, USA) and furfural and hydroxymethylfurfural were analyzed (20 µl) in an HPLC system, as described above. A reversed-phase Shimpack CLC-ODS column (150 mm×4.6 mm, 5 µm) was used at 30°C with an equivalent pre-column. The mobile phase consisted of methanol: 4% acetic acid (10∶90, v/v), with a flow rate of 1.0 ml min^−1^ for an isocratic run of 30 min. UV detection was performed at 280 nm. Furfurals were identified by comparing the retention times with the standard compounds. The furfurals content was calculated in relation to the lignin content obtained by the acetyl bromide method. Results were expressed as mmol L^−1^ furfurals g^−1^ cell wall.

### Quantification of lignin in the residues obtained from the thioglycolic acid method

In order to determine the content of residual lignin in the pellet discarded after the extraction of lignin by the thioglycolic acid method, an additional extraction of lignin was made using the acetyl bromide method (ε = 22.9 cm^−1 ^mg ml^−1^), as described above. The residues obtained were determined by measuring the absorption at 280 nm and calculated using the standard thioglycolic acid lignin (ε = 18.31 cm^−1 ^mg ml^−1^). Lignin contents were expressed as mg g^−1^ cell wall.

### Statistical design

The data are expressed as the mean of three or six independent experiments ± standard error (SE). The one-way variance analysis was performed to test the significance of the observed differences using the Sisvar package (Version 4.6, UFLA, Brazil). The differences between the parameters were evaluated by means of the Scott-Knott test. P values≤0.05 were considered as statistically significant.

## Results

### The lignin content as determined by the three analytical methods

Figure 1 shows the lignin contents in sugarcane bagasse, soybean root, and soybean seed coat as determined by the acetyl bromide, Klason, and thioglycolic acid methods. In sugarcane bagasse, the lignin contents determined by each method were 266, 242, and 28 mg g^−1^ cell wall by the acetyl bromide, Klason, and thioglycolic acid methods, respectively. Acetyl bromide method produced the highest recover of lignin, but was quite similar to the value of Klason, whereas the thioglycolic acid method provided the lowest recovery of lignin. In the soybean root, the lignin contents were determined to be 64, 66, and 21 mg g^−1^ cell wall by the acetyl bromide, Klason, and thioglycolic acid methods, respectively. Again acetyl bromide method provided a value for the lignin content similar that of Klason and thioglycolic acid method recovery the lower amount of lignin. In soybean seed coat the lignin contents were determined to be 45, 22, and 2.5 mg g^−1^ cell wall by the acetyl bromide, Klason, and thioglycolic acid methods, respectively. Here the recovery of lignin by Klason method was quite lower than that obtained with the acetyl bromide method. The thioglycolic method presented the lowest recover of lignin. Despite of these differences among them, all methods presented high reproducibility, as can be observed by the low standard errors (<5%).

### The lignin monomer cmposition

To evaluate whether the contrasting lignin contents obtained by the three methods were related to the lignin monomer composition, the contents of the H, G, and S units were determined by alkaline nitrobenzene oxidation (Table 1). The sugarcane bagasse lignin presented similar contents of G and S monomers and a minor content of H monomer, with an H:G:S ratio of 27∶35∶38. The soybean root lignin consists of a high content of the G unit when compared with the levels of the H and S units, and had an H:G:S ratio of 16∶69∶15. The lignin content in the soybean seed coat was formed by similar levels of the H and G units and a low content of S unit, with an H:G:S ratio of 44∶44∶12 (Table 1). Although nitrobenzene oxidation is not indicated for quantification the monolignol measurement allows an additional estimative of the relative content of lignin in the tissues through the sum H+G+S. The lignin abundance exhibited the following trend: sugarcane bagasse > soybean root > soybean seed coat.

**Table 1:** Lignin monomer composition of sugarcane bagasse, soybean root and soybean seed coat.

Sample	H	G	S	H+G+S	H:G:S
	mg g^−1^ cell wall				%
Sugarcane bagasse	4.35±0.19 b	5.75±0.36 a	6.23±0.27 a	16.33±0.76	27∶35∶38
Soybean root	0.15±0.01 b	0.64±0.01 a	0.14±0.01 b	0.93±0.02	16∶69∶15
Soybean seed coat	0.24±0.01 a	0.23±0.01 a	0.07±0.002 b	0.54±0.01	44∶44∶12

H, *p*-hydroxyphenyl; G, guaiacyl; and S, syringyl monomers. Mean values ± SE (N = 6) marked with different letters, in lines, are significantly different (P≤0.05, Scott-Knott test).

### The production furfural and hydroxymethylfurfural

**Figure 2:**
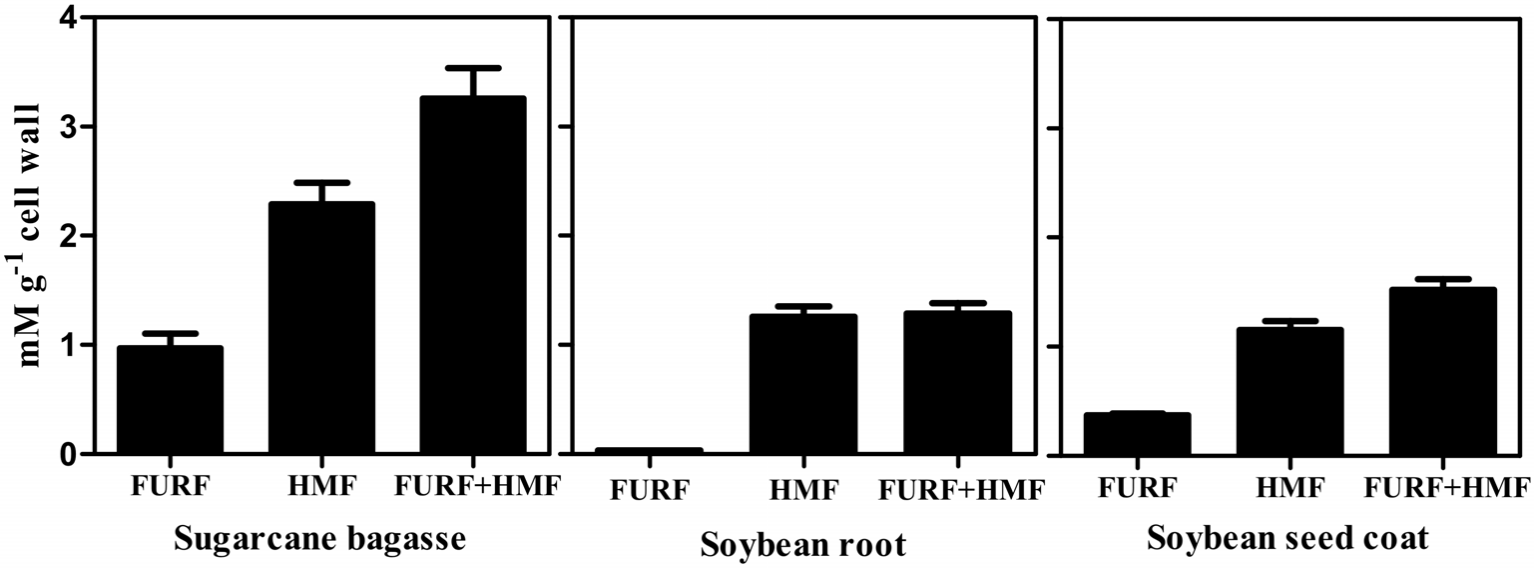
Furfural and hydroxymethylfurfural contents in sugarcane bagasse, soybean root and soybean seed coat determined after the acetyl bromide assay. Mean values ± SE (N = 3).

A possible oxidative degradation of structural polysaccharides can occur during the reaction with acetyl bromide by producing furfurals which are quantified together with lignin since it absorbs light at 280 nm [10]. So, furfural and hydroxymethylfurfural contents were determined in all samples after digestion with acetyl bromide (Fig. 2). The production of furfural and hydroxymethylfurfural was followed the trend: sugarcane bagasse > soybean seed coat > soybean root. However the percentage of interference was inversely proportional to the lignin content of the material evaluated.

### The lignin content in residues discarded by the thioglycolic acid method

While the absorptivities from acetyl bromide and thioglycolic acid methods are rather similar (see methods) the amount of lignin determined by the second was just a fraction of the first (Fig. 1). Therefore, the only possible explanation was that the thioglycolic acid procedure was not able to extract completely the lignin. So, we quantified the lignin present at the residual fractions that are discarded during the protocol procedures (Table 2). After the reaction of thioglycolic acid with lignin in a sample the lignin-thioglycolic acid complex (LTGA) is extracted with a solution of NaOH and the solid material remaining is discarded after centrifugation (see methods). By determining the content of lignin in this residue we discovered that most of lignin was still there. The lignin content as determined by thioglycolic acid method is presented in Table 2 as thioglycolic acid standard. Note that in bagasse, the content of lignin in the residue is almost the same determined in the standard. In the other tissues, most of lignin remained in the residue reaching more then 10 times the standard in soybean coat seed. We also determined the lignin contents in the supernatant discarded after the precipitation of LTGA with HCl (see methods). Only a small amount of lignin was present when compared to the values of thioglycolic acid standards.

**Table 2:** Lignin concentration obtained from thioglycolic acid residues.

Method	Sugarcane bagasse	Soybean root	Soybean coat
	mg.g^−1^ cell wall		
Acetyl Bromide (solid residue)	15.98±0.29	29.42±0.96	19.28±1.10
Acid step of TGA	0.27±0.01	0.11±0.01	0.06±0.00
TGA	28.05±0.94	21.25±0.77	2.46±0.94
TOTAL	50.630	62.44	29.44

Mean ± SE values (N = 3).

## Discussion

### Causes of thioglycolic low performance

The thioglycolic acid method has been considered the most suitable for the isolation and quantification of lignin in herbaceous plants [6], although it is known that it can underestimate the lignin levels due to the high specificity of the reaction with ether bonds of lignin [11]. The β–O–4 is the main linkage connecting propenyl chains of monolignols [36] but, there are other linkages that do not react with thioglycolic acid. Thus, the specificity could prevent a complete release of lignin into the alkaline solution and lignin fragments can be lost as solid residues discarded. Indeed, our data showed low amounts of thioglycolic lignin in all plant materials assayed (Fig. 1). Parameters as detection and quantification limits are similar for lignin solubilized either by thioglycolic or acetyl bromide methods. Indeed, standard curves of both thioglycolic (ε = 18.31 g^−1 ^L cm^−1^) and acetyl bromide (ε = 22.9 g^−1 ^L cm^−1^) were close. So, given the remarkable lower recovery of lignin presented by thioglycolic acid method we investigated the possible losses of lignin during the assay. For this, we used the acetyl bromide method to determine the presence of lignin in the residues produced by the thioglycolic method. As shown in Table 2, the solid residue from the thioglycolic acid reaction had a very high residual content of lignin. For the root and seed coat of soybeans, these values were even higher than those obtained by the thioglycolic acid standard. This explains, at least in part, the reason why the values of lignin determined by the thioglycolic acid method were low (Fig. 1). In addition, the thioglycolic acid method has many washing steps, and some lignin could be discarded. Besides, it was suggested a possible solubilization of lignin during the acid step [9]. In order to investigate eventual disposal of lignin in washing steps, we measured the absorbance at 280 nm of all residues from the acid step, and calculated the corresponding lignin contents. We found that the lignin content of each soluble residue was rather small when compared with the thioglycolic acid standard (Table 2). However, the sum of the LTGA plus the lignin wasted in the thioglycolic acid protocol (reconstituted-LTGA) was comparable to the value of Klason lignin in both type I cell wall (soybean) tissues. On the other hand, in sugarcane bagasse, both LTGA and reconstituted-LTGA failed completely, while the Klason and acetyl bromide methods gave similar results.

The acetyl bromide method gave the best recovery of lignin in all samples evaluated. Using acetyl bromide and Klason protocol, we showed that sugarcane bagasse presented the highest lignin content, followed by soybean root and seed coat (Fig. 1). Lignin content varies during the stages of vegetative growth, with high levels being present during maturity [25]. The values of lignin content determined by the acetyl bromide and Klason methods were similar in sugarcane bagasse, a mature, lignified tissue. These results are similar with others reported in literature [13], [14]. The content of lignin in soybean roots, as determined by acetyl bromide and Klason methods, were also similar. However, the lignin content presented by acetyl bromide and Klason were discrepant in the less lignified tissue (Fig. 1). Thioglycolic assay gave the lowest values among all the three tested methods confirming that each method can give different lignin values for the same type of tissue [9].

### Causes of the discrepancy between Klason and acetyl bromide methods

The contrasting results produced by Klason and acetyl bromide methods in seed coat together but close results in the other tissues suggest that the accuracy of one of the two techniques is tissue-dependent. In order to investigate such tissue-dependent performance we assessed three hypotheses: 1) monolignol composition interference, 2) sensitivity to lignin content and 3) furans production.

1. The main constituents of lignin are the H, G, and S monomers, which are linked by β–O–4-ether bonds into the polymer [26] whose proportion can vary between plant species, tissues of the same plant, and different layers of cell wall [27], [28]. The monomer composition might determine the intensity of cross-linkages among the units [29] and it can interfere with the amount of lignin solubilized by each extraction protocol. In order to investigate the influence of monomer composition in the methods efficiency in recover lignin we determined the levels of the H, G, and S unities [30]. The H+G+S sum is a reasonable estimate of the condensation degree of the lignin polymer [31]. The lignin monomer composition was different among the tissues evaluated (Table 1). The values of H+G+S for the soybean root and seed coat were in agreement with the high lignin values determined by the acetyl bromide and Klason methods (Fig. 1). While the H:G:S ratio for the soybean root was typical of angiosperms (*i.e.* G>H = S), the soybean seed coat ratio presented the unusual ratio of H = G>S. Nevertheless, the amounts of H+G+S for both tissues were in agreement with values of total lignin (*i.e.* root > seed coat; Fig. 1). As expected for the type II cell walls, the H:G:S ratio for sugarcane bagasse was S>G>H [32], [33]. However, despite of the different monomeric ratios, no correlation between monolignol composition and the contrasting results of the methods were noted.
2. The second hypothesis evaluated was that the Klason method looses accuracy in tissues containing very low lignin content. Lignin can be divided in *core* and *non-core* lignin. *Core* lignin consists of highly condensed polymeric matrices, while *non-core* contains low molecular weight phenolic monomers and oligomers. The ratio of *core*/*non-core* lignin decreases in tissues containing less lignin and the Klason method can be used to extract only *core* lignin [34] while the acetyl bromide method can be used to quantify both *core* and *non-core* lignin [35]. The discounted contribution of ash in Klason method can not explain the differences observed. As can be seen in Table 3, the ash contribution for the insoluble lignin was less than 5% in all the three biomasses. Although the proportion of ash lignin has varied among the tissues, these 5% are far from explain the differences observed between acetyl bromide and Klason methods found in soybean seed coat which was of about 50%.
3. A third hypothesis assessed was that the acetyl bromide method overestimates lignin content due to the formation of furfurals in tissues with higher content of polysaccharides. The combination of high temperature plus low pH used in acetyl bromide method can produce furan derivatives, e.g. furfural, hydroxymethylfurfural, and methoxymethylfurfural. Furans results from dehydration of monosaccharides and absorb light at 280 nm, which could result in an overestimation of the lignin content [10]. The interference of furans to Klason lignin was negligible. The soluble lignin fraction where furans could be produced contributed with less than 12% of total Klason lignin. Even so, we applied the Goldschmidt’s equation, which discounts interference of furfurals [23] to calculate the soluble lignin in the acid medium used in the Klason method. The interference of furfurals produced by the acetyl bromide method was lower than 10% in all tissues evaluated. However, the percentage of furfurals interference was inversely proportional to the amount of lignin of the tissue. Thus, might be necessary to consider the interference of furfurals in tissues with very low lignin content. Such interference, however, can not explain the differences in lignin content observed between Klason and acetyl bromide methods. Together these evidences suggest that Klason method loses efficiency in lignin recovery in plant materials poor in lignin.

**Table 3:** Contributions of ash, insoluble and soluble lignin concentration for total Klason lignin.

Sample	Acid-insoluble ash	Insolublelignin	Soluble lignin	Total lignin
	mg g^−1^ cell wall			
Sugarcane bagasse	8.3±0.21	227.07±2.27	14.30±0.88	241.4±1.85
Soybean root	2.8±0.18	58.23±2.13	7.73±0.12	65.96±0.89
Soybean seed coat	0.5±0.06	21.38±0.79	0.32±0.03	22.70±0.30

This low ability of Klason method to recover lignin in tissue with low lignin content was the unique bias observable among the methods. Otherwise, all methods presented high reproducibility in all tissues assessed. On the other hand, the acetyl bromide method presented higher recovery of lignin when compared with the other methods in all tissues evaluated. Besides, acetyl bromide method is simpler and faster than the other methods tested. This way, we suggest that among the techniques assessed in this study, the acetyl bromide is a preferable methodology to determine lignin content in herbaceous tissues.
